# Anti-Candidal Activity and In Vitro Cytotoxicity Assessment of Graphene Nanoplatelets Decorated with Zinc Oxide Nanorods

**DOI:** 10.3390/nano8100752

**Published:** 2018-09-21

**Authors:** Graziella Ficociello, Maria Giovanna De Caris, Giusy Trillò, Domenico Cavallini, Maria Sabrina Sarto, Daniela Uccelletti, Patrizia Mancini

**Affiliations:** 1 Department of Biology and Biotechnology, Sapienza University of Rome, Piazzale Aldo Moro 5, 00185 Rome, Italy; graziella.ficociello@uniroma1.it (G.F.); gtgiusy.92@hotmail.it (G.T.); 2 Department of Experimental Medicine, Sapienza University of Rome, Viale Regina Elena 324, 00161 Rome, Italy; mariagiovanna.decaris@uniroma1.it (M.G.D.C); patrizia.mancini@uniroma1.it (P.M.); 3 Department of Aerospace, Electrical and Energy Engineering, Sapienza University of Rome, Via Eudossiana 18, 00184 Rome, Italy; domenico.cavallini@uniroma1.it (D.C.); mariasabrina.sarto@uniroma1.it (M.S.S.); 4 Research Center for Nanotechnology Applied to Engineering of Sapienza University (CNIS), Sapienza University of Rome, Piazzale Aldo Moro 5, 00185 Rome, Italy; 5 Sapienza Nanotechnology & Nano-science Laboratory (SNN Lab), Sapienza University of Rome, Piazzale Aldo Moro 5, 00185 Rome, Italy

**Keywords:** *Candida albicans*, biofilm, zinc oxide, nanorods, graphene, HaCaT cells

## Abstract

*Candida albicans* is the most common pathogenic fungus that is isolated in nosocomial infections in medically and immune-compromised patients. The ability of *C. albicans* to convert its form from yeast to hyphal morphology contributes to biofilm development that effectively shelters *Candida* against the action of antifungals molecules. In the last years, nanocomposites are the most promising solutions against drug-resistant microorganisms. The aim of this study was to investigate the antifungal activity of graphene nanoplateles decorated with zinc oxide nanorods (ZNGs) against the human pathogen *Candida albicans*. We observed that ZNGs were able to induce a significant mortality in fungal cells, as well as to affect the main virulence factors of this fungus or rather the hyphal development and biofilm formation. Reactive Oxygen Species (ROS) formation in yeast cells resulted one of the mechanisms of ZNGs to induce mortality. Finally, the toxicity of this nanomaterial was tested also on human keratinocyte cell line HaCaT. Our data indicated that ZNGs resulted not toxic when their aggregation state decreased by adding glycerol as emulsifier to ZNGs suspensions or when HaCaT cells were grown on ZNGs-coated glasses. Overall, the results that were obtained indicated that ZNGs could be exploited as an antifungal nanomaterial with a high degree of biocompatibility on human cells.

## 1. Introduction

The worldwide incidence of infectious diseases continues to increase, and causative microorganisms are increasingly showing (multi-)drug resistance.

Microorganisms such as *Escherichia coli*, *Staphylococcus aureus*, and *Candida albicans* are part of the human microbiota. However, pathogenic forms of these microbes have been implicated in blood or urinary tract infections, gastroenteritis, endocarditis, soft tissue infections, and organ malfunction [1].

The common pathogenic fungus, *C. albicans* is a commensal organism in the mucocutaneous cavities of the skin, vagina, and intestine of humans. However, when the host immune defense system become weakened it can cause infections. This fungus represents the fourth-greatest cause of bloodstream infections and together with other opportunistic pathogens that are commonly colonized the human oral mucosa [2]. *C. albicans* is the most widespread yeast pathogen and is responsible for 50% of candidiasis, with a mortality rates in nosocomial infection reaching half of infected patients [3]. The pathogenicity of *Candida* species depends on a variety of determinants such as adhesion factors, germ tube and hyphal and biofilm formation [4]. The germ tube formation represents the transition from a budding state to hyphal cells and it is known to facilitate yeast adherence to epithelial cells and promote the aggregation of yeast cells [5].

Most of antifungal drugs used to treat *C. albicans* infections target the synthesis and function of ergosterol, which is a major component of the fungal cell membrane. The efficacy of these drugs is compromised by the emergence of resistant strains and the ability of this fungus to form biofilm [6]. Therefore, it is urgent to develop novel non-toxic and efficient agents and strategies against this opportunistic pathogen. Due to the outbreak of the infectious diseases that are caused by different pathogenic microorganism, during the last years, a great importance was given to the research of novel compounds with antimicrobial activity. In this regard, nanomaterials and nanoscience seem to be a good solution in solving this public health problem. Nanotechnologies are widely used in a number of processes, like material science, agricultural production, food industry, cosmetic, as well as sophisticated medicinal techniques (reviewed in [7,8]). Furthermore, nanomaterials have attracted tremendous interest in the fields of photocatalysis, sensors, solar cells, and supercapacitors [9,10]. Nanotechnology deals with the manufacture and application of materials with size of up to 100 nm. Inorganic nanoparticles exhibit significantly novel and distinct physical, chemical, and biological properties, whose have elicited much interest over the past few decades because of their potential for biological and pharmaceutical applications [11,12,13]. In this scenario, the use of inorganic nanoscale materials has been increased also as novel antimicrobial agents, owing to their high surface area relative to volume ratio and the unique physical and chemical properties [14]. In addition, these materials are also more stable at high temperature and pressure [15]. Among these nanomaterials, it has been reported that metallic nanoparticles and metal oxide nanoparticles, such as aluminum oxide (Al_2_O_3_), silicon dioxide (SiO_2_), titanium dioxide (TiO_2_), and zinc oxide (ZnO), display the most antibacterial properties [15]. Particularly, zinc oxide shows toxic activity over a wide spectrum of bacterial species [16,17,18]. ZnO is biocompatible, non-toxic, photochemically stable, as well as it has also been indicated as a generally recognized as safe (GRAS) material by the U.S. Food and Drug Administration (21CFR182.8991) [19]. It shows bactericidal properties over a broad range of Gram-positive as well as Gram-negative bacteria, like *Escherichia coli*, *Streptococcus pyogenes*, *Bacillus subtilis*, *Staphylococcus aureus*, *Salmonella enteritidis*, *Listeria monocytogenes*, *Klebsiella pneumonia*, *Pseudomonas aeruginosa*, *Salmonella typhimurium*, *Enterococcus faecalis*, etc. (reviewed in [20]). Nano-sized ZnO exhibits varying morphologies, depending on the process of synthesis. For example, they may be nanorods, nanoplates [21,22], nanowires, nanotubes [23,24], nanospheres [25], nanorings [26], and nanoboxes [27]. ZnO nanoparticles exhibited also significant antifungal properties against important plant pathogenic fungi, such as *Botrytis cinerea* and *Penicillium expansum* [28]. Moreover, ZnO nanorod arrays are able to diminish the growth of human opportunistic fungus *Candida albicans* with stable action for two months [29].

Although there are several advantages of using nanomaterials, the widespread application of these materials confer enormous potential for human exposure and environmental release [30], thus, a better understanding of the toxicological aspects on nanomaterials is essential [31].

The general properties of nanomaterials regarding risk assessment are dependent on the essential characteristics of the nanostructures that are directly related to their synthesis methods [32].

Several nanomaterials with different physical-chemical properties resulted in being permeable to skin. The skin in fact, could serve as an important door for the entry of nanoparticles in the human body [33]. Cytotoxicity can be evaluated in the human adult low calcium high temperature (HaCaT) cell line, since this is derived from adult human skin that exhibits normal differentiation capacity [34,35]. HaCaT cells are also regarded as a promising alternative approach that is designed to mimic in vivo models in humans as closely as possible [36,37]. Recently, low toxicity of zinc oxide nanorods and microrods has been reported against two different human cell lines: the breast cancer cells MCF7 and the immortalized keratinocytes HaCaT [38]. Moreover, ZnO-NPs are reported as non-toxic to human cells in several studies allowing for their use as antibacterial agents [39,40].

This study investigated the anti-*Candida* properties of a hybrid nanomaterial made up of graphene nanoplatelets decorated with ZnO nanorods (ZNGs). We analysed its toxic effect on fungal vitality as well as on *C. albicans* virulence factors like the hyphal development and biofilm formation. Furthermore, the biocompatibility of ZNGs was assessed while utilizing in vitro tests on HaCaT cells.

## 2. Results and Discussion

### 2.1. ZNGs Exert Antifungal Activity in C. albicans Cells

Previously, it was demonstrated that zinc oxide nanorods decorated graphene nanoplatelets exert a higher antimicrobial activity against both the gram-negative *Pseudomonas aeuruginosa* and the gram-positive *Staphylococcus aureus* bacteria, responsible for nosocomial infections as well as for cultural heritage deterioration [41]. Moreover, ZnO-NRs decorated GNPs severely affect the cell vitality of cariogenic bacterium *Streptococcus mutans* [42].

Thus, we decided to investigate whether this hybrid nanomaterial could also have an anti-fungal effect against the opportunistic pathogen *C. albicans*.

**Figure 1:**
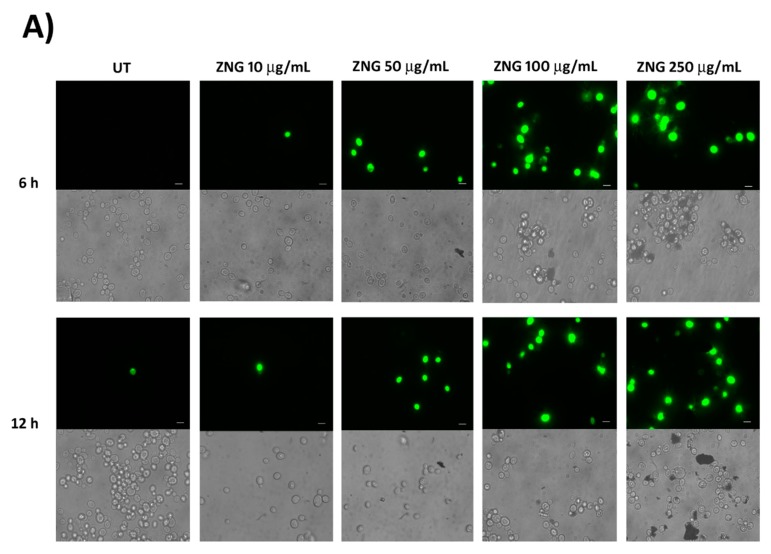

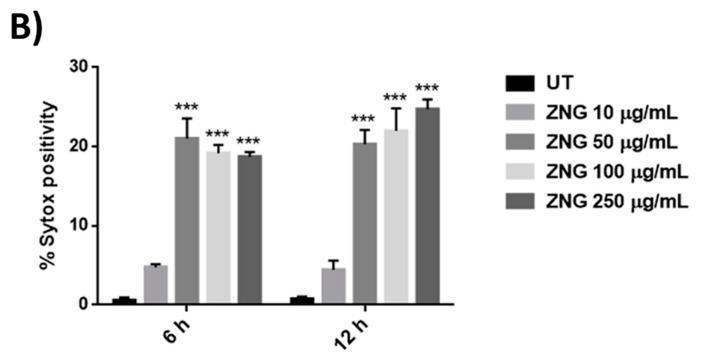
Viability of *C. albicans* treated with ZnO nanorods (ZNGs) material. (**A**) SYTOX Green staining of *C. albicans* cells exposed or not to ZNGs for 6 h or 12 h. Scale bar 5 µm; (**B**) Percentage of fluorescent died cells relative to the total cells. The data were analyzed by two-way analysis of variance (ANOVA), followed by Bonferroni’s test. (*** *p* < 0.001).

We first analysed the ZNGs ability to induce *C. albicans* cell death. For this purpose, we employed the non-cell permeable DNA dye SYTOX Green. Indeed, this fluorescent dye is able to penetrate only the dying cells. Yeast cells were exposed for 6 h and 12 h to increasing concentrations of ZNGs and is then stained with SYTOX Green. A shown in Figure 1A,B, although ZNGs were not effective at the lowest concentration of 10 µg/mL, a noteworthy fungal mortality was highlighted when ZNGs concentration was increased up to 250 μg/mL (Figure 1A). The SYTOX Green positive cells rose to ~ 20% both after 6 h and 12 h of ZNGs treatment at 50 μg/mL (Figure 1B).

**Figure 2:**
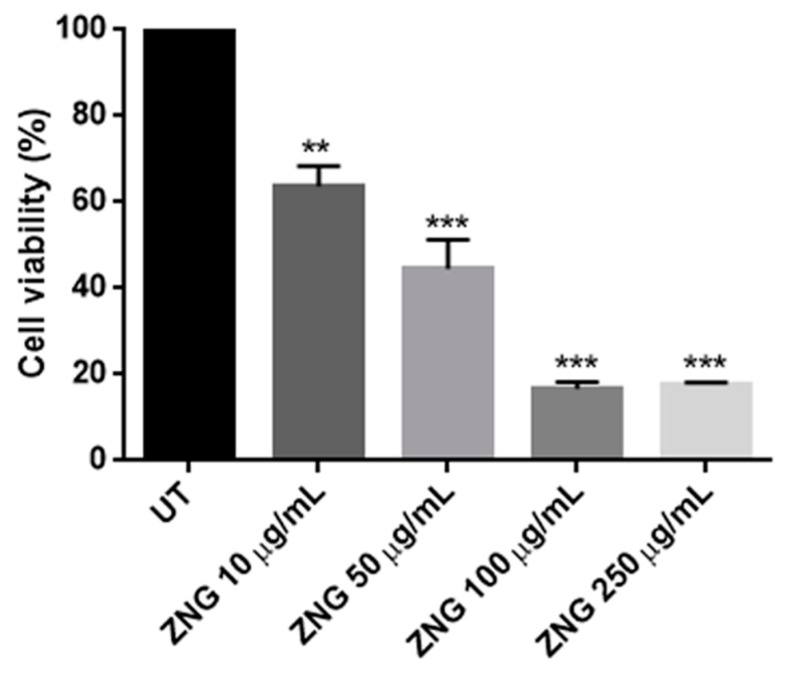
Analysis of Colony Forming Units (CFU) counting for *Candida albicans* cells untreated (UT) or treated with ZNG nanomaterials at different concentrations. Statistically significant differences with the untreated group were calculated by using one-way ANOVA with Bonferroni’s post-test. (** *p* < 0.01, *** *p* < 0.001).

To confirm this data, *C. albicans* cells were incubated for 24 h with different concentrations of ZNGs and then the Colony Forming Units (CFU) assay was performed. After a long-term exposure, also 10 μg/mL ZNGs induced a slight mortality of *C. albicans*, as long as the cell survival was decreased by 50% when fungal cells were exposed to 50 μg/mL ZNGs. However, the lowest cell viability was achieved at the two higher concentrations with a mortality rate of about 80% (Figure 2).

Based on these results ZNGs displayed a significantly antifungal effect against the opportunistic fungus *C. albicans*. In addition, zinc oxide nanoparticles were shown to induce a reduction of growth rate, as well as the cell viability of *C. albicans*, with a dose-effect response that was very similar to ZNGs [43].

It was reported that ZnO nanorods that are vertically aligned on a glass surface exert an inhibitory action on *C. albicans* growth [29]. This is probably due, as in the case of ZNGs, to the increased surface of the nanomaterial that enhanced the contact between ZnO nanorods and the cells.

### 2.2. ZNGs Induce Intracellular ROS Accumulation

**Figure 3:**
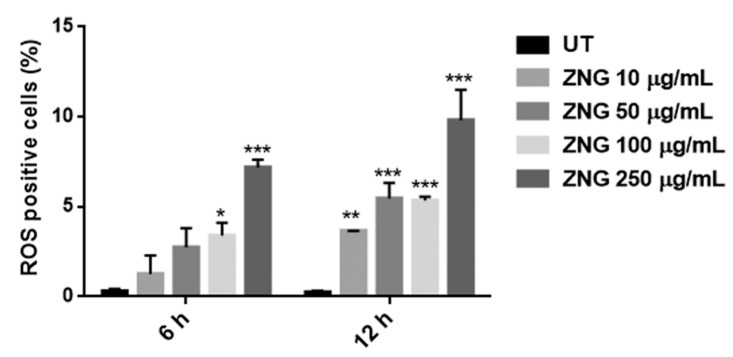
Detection of ROS content using H_2_DCFDA staining. *C. albicans* cells untreated (UT) or treated with the various concentrations of ZNGs, for 6 h and 12 h, were stained to detect the intracellular ROS formation. The bars represent the percentage of fluorescence-positive cells with respect to total cells. Means and SDs are shown (* *p* < 0.05, ** *p* < 0.01, and *** *p* < 0.001 when compared with the untreated cells).

Oxidative stress mediated by ROS is a well-known inducer of cytotoxicity and cell death in *C. albicans* [44]. Therefore, we examined the possible role of ROS in anti-candidal activity of ZNGs. To this aim, we used the cell permeable dye H_2_DCFDA that, when binding the intracellular ROS, is rapidly oxidized into the highly fluorescent DCF [45]. *C. albicans* cells were exposed with the increasing ZNGs concentrations for 6 h and 12 h. As shown in Figure 3, the hybrid material at the concentrations of 100 and 250 μg/mL induced ROS accumulation in both a time- and dose-dependent manner, meanwhile the two lowest concentrations were able to induce ROS accumulation only after 12 h of exposure.

In the light of these results, we supposed that the antifungal effect of ZNGs against *C. albicans* was due to the increased ROS levels. It has been reported that free radicals that are produced by ZnO nanoparticles in water suspensions are directly correlated with an increase of ROS productions, as well as a reduction of cell viability in *C. albicans* cells [43].

However, for zinc oxide-based nanomaterials, two mechanisms have been proposed for their antimicrobial action. The first is that ZnO in aqueous solution generates hydrogen peroxide (H_2_O_2_) from its surface, that can penetrate the cell membrane of microorganisms and kill them, inducing oxidative stress. The second one is the release of zinc ions in medium with effects on active transport as well as on the amino acid metabolism [46]. Since we previously showed that the release of zinc ions by ZNGs in the medium is very low [42] we investigated if the main mechanism of toxicity against *C. albicans* was due to the production of H_2_O_2_, that causes the oxidative stress in fungal cells. To assess this hypothesis, we performed the cell vitality assay in the presence of 10 mM histidine, which is a quencher of hydroxyl radicals and singlet oxygen [43]. The effect of histidine on ZNGs toxicity was tested by adding this scavenger to fungal cultures containing different concentrations of nanomaterials. We found that histidine was able to recover the *C. albicans* viability only partially, with the cell survival percentage raised from 47% to 62% in the case of cells that were treated with 50 µg/mL ZNGs, while a cell survival from 15% to 55% was observed when the two highest concentrations of ZNGs were utilized (data not shown). These results indicate that the toxicity exerted by ZNGs on *C. albicans* cells is probably partly due to the intracellular ROS accumulation and partly to mechanical damages that are caused by ZnO-NRs that act as nano-needles.

### 2.3. Hyphal Development Is Affected by ZNGs Treatment

The transition from yeast to hyphal form is essential for the pathogenicity of this opportunistic fungus [47]. Indeed, while the yeast morphology plays a key role in the dissemination and systemic infection, the hyphal development is decisive for the biofilm formation and it is important to establish and maintain the infection [48]. Moreover, the germ tubes also give resistance against the phagocytosis from immune system cells when compared with the budding form [49].

**Figure 4:**
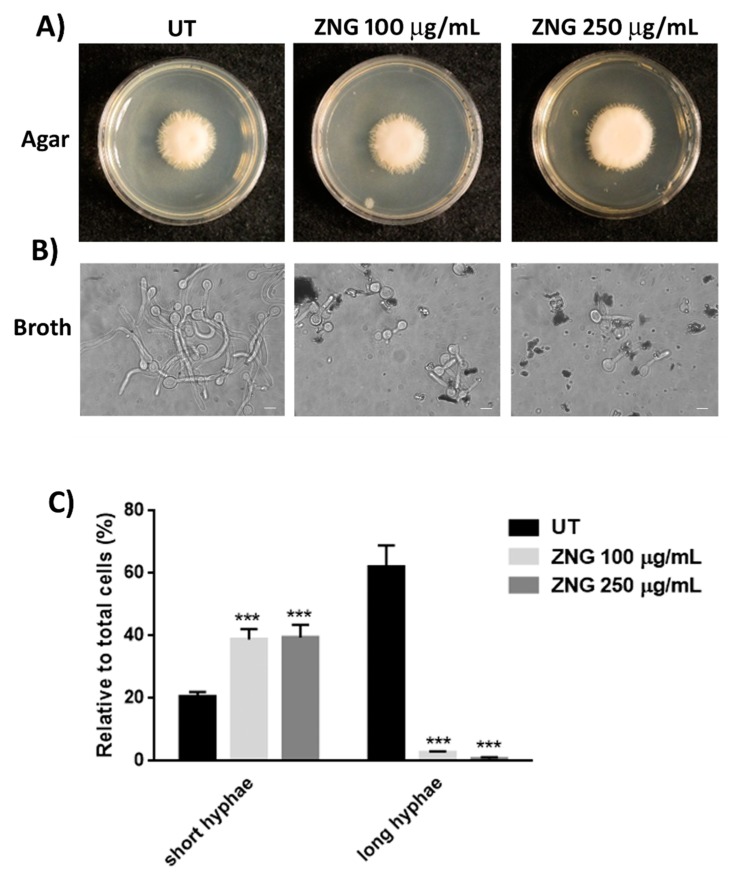
Inhibition of hyphal formation by ZNGs. (**A**) Effect of 100 µg/mL and 250 µg/mL ZNGs on *C. albicans* hyphal elongation on solid Spider medium. Plates were incubated at 37 °C for five days; (**B**) ZNGs activity on *C. albicans* hyphal formation in liquid Spider medium for 2.5 h at 37 °C. Scale bar 5 µm; and, (**C**) Percentage of short and long hyphae observed after 2.5 h of treatment with ZNGs. Untreated cells (UT) were used as control. A two-way ANOVA analysis with the Bonferroni post-test was used to assess the statistical significance (*** *p* < 0.001 with respect to untreated cells).

For this reason, we next examined the effect of ZNGs on *C. albicans* hyphal elongation on solid medium by cultivating fungal cells and observing colony morphology on Spider agar supplemented with the various concentrations of ZNGs at 37 °C (Figure 4A). This hybrid nanomaterial exerted a slight effect at the concentration of 100 µg/mL, however, it found a strong reduction of filamentation when *C. albicans* was treated with the highest concentration of ZNGs (Figure 4A). We also examined the impact of ZNGs on hyphal growth in liquid Spider media. In this case, *C. albicans* cells that were treated with 100 µg/mL or 250 µg/mL ZNGs showed a considerable arrest of the hyphal development (Figure 4B), with a decrease of long hyphae of 96% and 99%, respectively, when compared to the untreated sample (Figure 4C). Conversely, the exposure to the lowest concentrations of the hybrid nanomaterials was not able to alter the hyphal morphology, both on Spider agar and in liquid medium (data not shown).

Similarly to our findings, it was showed that silver nanoparticle decorated with hydroxyapatite are able to reduce the hyphal cell density of *C. albicans* cells, although they were exposed to high concentrations of this nanomaterial [50]. Moreover, biogenic silver nanoparticles synthetized while using the aqueous leaf extract of *Polyalthia longifolia* significantly reduce the serum-induced hyphal form morphogenesis in *C. albicans* [51]. Recently, it was reported that nanoparticles induce the formation of pseudo-hyphae, an index of yeast cellular stress [52]. Currently, few works describe the effects of graphene- or zinc oxide-based nanomaterials on hyphal formation of *C. albicans*. It is reported that ZnO-NPs synthetized from leaf extract of *Crinum latifolium* are able to inhibit the hyphal development. This nanomaterial induces a reduction of germ tube formation of 34% when it is used at the concentration of 250 µg/mL [53]. We previously found that ZNGs induce mechanical injuries against the bacteria *S. aureus* and *P. aeruginosa*. The ZnO-NRs protrude from the GNPs acting as nanoneedles that pierce the cell wall. Moreover, the GNPs platelets could offer a large surface that hampers the hyphal development.

### 2.4. ZNGs Strongly Reduce Cell Viability in C. Albicans Biofilm

The ability of *Candida* spp. to form biofilms on medical devices, such as catheters, contact lenses, dentures, and voice prostheses represents another healthcare problem that is associated with this pathogenic fungus [54,55]. The capability of *C. albicans* to form biofilm is the major factor that contributes to its high resistance to antifungal drugs [56,57] and Candida biofilm associated with medical devices tend to be more problematic to eradicate than bacterial infections [58]. Indeed, *C. albicans* resistance is reported for all of the antifungal drugs that have been widely deployed in therapy [59].

Thus, there is an urgent need for novel strategies for the prevention and treatment of *C. albicans* biofilm development.

**Figure 5:**
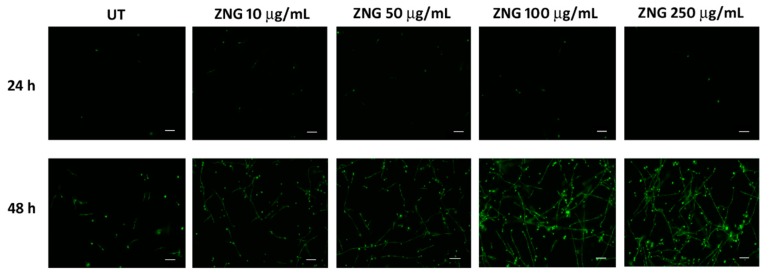
Fungicidal activity of ZNGs nanomaterials on *C. albicans* biofilm. The biofilm formation, at the indicated concentrations of ZNGs, was obtained in 12-well cell culture plates at 37 °C for 24 h or 48 h. Afterward, the cells were stained with SYTOX Green and visualized at 32× magnification. Untreated biofilm (UT) was used as negative control. Scale bar 20 µm.

In this work, we assessed the toxicological capability of ZNGs on *C. albicans* biofilm, while using a model of biofilm formation on the 12-wells microtiter plates. Fungal cells were grown for 24 h or 48 h, in the presence of the increasing concentrations of ZNGs in the appropriate conditions to induce biofilm formation. We observed that ZNGs were not able to prevent the biofilm development in the treated wells when compared to the control. Furthermore, optic microscope observations revealed that the cell adhesion was not altered by ZNGs exposure. Afterwards, we stained mature biofilms with the SYTOX Green dye to detect the cell mortality. After 24 h of exposure in the presence of ZNGs, no differences in the cell vitality were observed between the control and treated samples. On the contrary, ZNGs exhibited a notable fungicidal effect on *C. albicans* biofilm after 48 h of treatment, already at the low concentration of 10 μg/mL (Figure 5).

As mentioned before, the ability to form biofilm is the main virulence factor of *C. albicans*. Thus, investigate the toxicity against the biofilm is important to develop novel nanomaterials with anticandidal activity. In literature, the inhibition of *C. albicans* biofilm by other nanoparticles has been reported, golden nanoparticles (AuNPs) significantly reduce the biofilm density of *C albicans* cells, although they resulted ineffective on yeast growth and hyphal development [60]. Biogenic selenium nanoparticles, as produced by reactions of reduction of environmental bacterial isolates, inhibit biofilm formation of 60–70%, without affecting the growth rate of this pathogenic fungus [61]. Zinc oxide nanomaterials coated or not with different molecules were tested for their ability to prevent the biofilm formation of *C. albicans*. For example, zinc oxide nanoparticles that were coated with biopolymer gelatin or chitosan-linoleic acid led to a weak adherence of biofilm formation [62,63]. Moreover, it was found that ZnO-NPs had a toxic effect on *C. albicans* biofilm inducing alterations in the extracellular matrix [53]. The effects on biofilm extracellular matrix were reported also for ZNGs that inhibit the exopolysaccharide (EPS) production of cariogenic bacterium *S. mutans* [42].

### 2.5. Biocompatibility of HaCaT Cells

**Figure 6:**
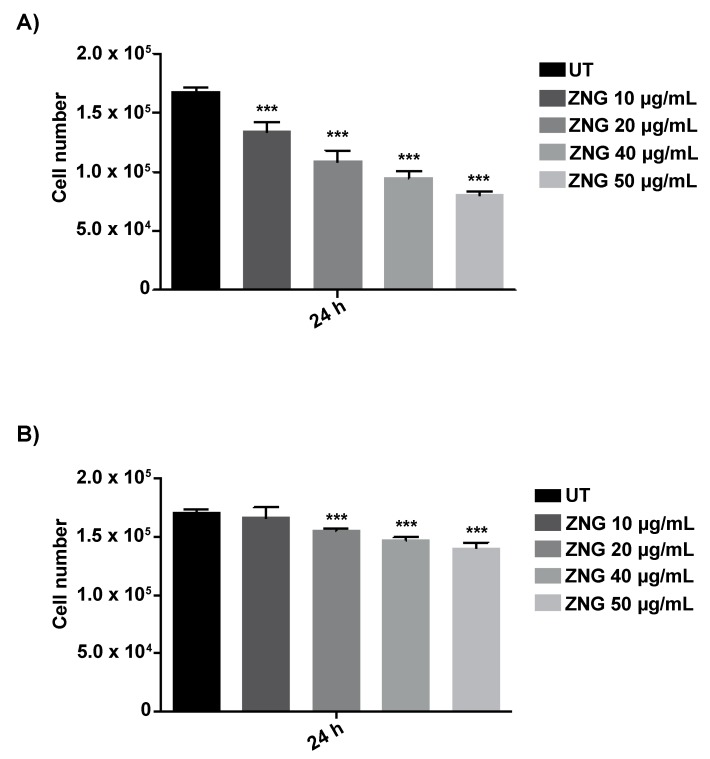
Cell viability of HaCaT cells after treatment with ZNGs suspended in (**A**) Dulbecco’s Modified Eagle’s Medium (DMEM) or (**B**) 4% glycerol in DMEM. The cell count was performed by the Trypan Blue dye exclusion test, and the values were reported as total number of living cells. Data are expressed as mean ± SD, *n* = 3. Statistical analysis was performed by one-way analysis of variance (ANOVA) method coupled with the Bonferroni post-test (*** *p* < 0.001 compared to the untreated sample (UT)).

Since we have previously demonstrated that the human cell lines HaCaT and MCF7 show a very low cytotoxicity when exposed to ZnO-NRs and ZnO-MRs [38], in this report, we wondered whether HaCaT cells had a low cytotoxicity also towards ZNGs. For this purpose, human keratinocytes were incubated with different concentrations of ZNGs suspended in culture medium (DMEM) for 24 h. Results obtained show that already at 10 µg/mL of ZNGs concentration a reduction of cell viability was noticeable, which became more evident at higher concentrations, indicating a dose-dependent cytotoxic effect of ZNGs (Figure 6A). We assume that this result may depend on the strong aggregation of ZNGs suspended in the culture medium observed at higher concentrations. As the cytotoxicity of a nanomaterial depends on its shape, size and aggregation state, it becomes essential to increase its biocompatibility and stability in different aqueous solutions or different synthesis preparation. For example, Harper and colleagues found that the toxicity of golden nanoparticles coated with Glutathione (Au-GSH NPs) was significantly reduced and the ligand structure was ameliorated when the ultracentrifugation purifications were implemented during the multi-step synthesis and surface modification of Au-GSH nanoparticles [64].

Moreover, biocompatibility can be enhanced by adding to the suspension of nanomaterials reagents, such as chitosan [65], bovine α-lactalbumin [66], or polyethylene glycol [67].

In this report, in order to reduce the cytotoxicity of ZNGs, we employed glycerol, a stabilizer of emulsions [68], which is generally used to increase the biocompatibility of different types of nanoparticles [69]. The incubation of HaCaT cells with ZNGs suspended in culture medium with the addition of 4% glycerol, led to a marked improvement in cell viability (Figure 6B). These results can be explained by the fact that glycerol could behave like an osmoprotector. Indeed, Corrales et al. reported that glycerol has an osmoprotective function in human corneal epithelial cells. They demonstrate that the addition of glycerol to medium of isotonic, physiologic osmolarity is responsible for the strong reduction of activation of Mitogen-activated protein kinases (MAPKs), which represents a marker of the osmolarity cell response [70].

For this reason, in the following experiments we used ZNGs dispersed in culture medium with 4% of glycerol.

### 2.6. Cell Proliferation and Cytoskeleton Morphology in HaCaT Cells

Since microtubules are highly dynamic components of the cytoskeleton, being essential for intracellular transport, organization of organelles and cell division [71], here we have analyzed cell proliferation and the distribution and morphology of tubulin, which is a fundamental component of microtubules.

**Figure 7:**
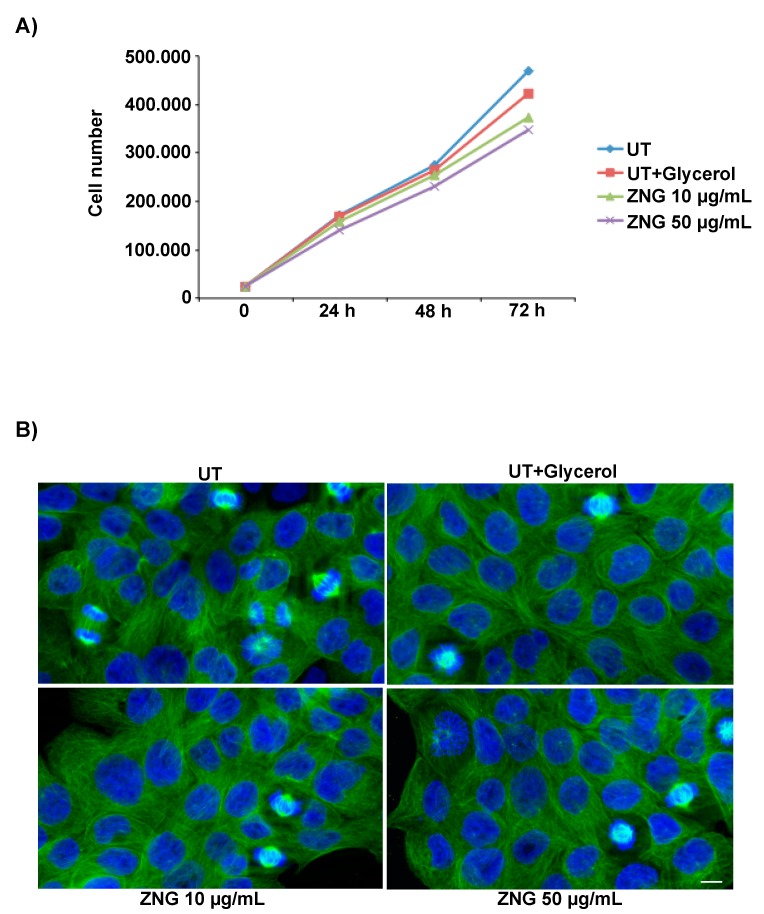
(**A**) Cell proliferation and (**B**) immunofluorescence analysis of FITC-tubulin staining of HaCaT cells after exposure to ZNGs. Scale bar represents 20 µm.

For cell proliferation, HaCaT cells were incubated or not with ZNGs for 24, 48 and 72 h. The results obtained show a normal proliferative trend (Figure 7A). In particular, the growth curves of the cells treated with 10 µg/mL or 50 µg/mL of ZNGs exhibited an exponential trend, similar to that of the control cells, although a very lower proliferation rate was present already at 24 hours in a dose-dependent manner (Figure 7A). These results indicate that the ZNGs do not impair the proliferation of HaCaT cells. For tubulin analysis, we performed an immunofluorescence study on HaCaT cells that were treated with ZNGs, as above. Fluorescence images show a normal localization and organization of the tubulin, in all the samples examined (Figure 7B). Moreover, well organized mitotic spindles are very evident, indicating that the ZNGs did not alter the normal distribution and morphology of microtubules, and that cells are in active proliferation rate (Figure 7B).

We also examined the actin cytoskeleton organization in cells that were treated with ZNGs. Immunofluorescence images show that actin was mainly arranged in filopodia (Figure S1), probably used by the cells as probes of the surrounding environment [72]. After treatment with 10 µg/mL or 50 µg/mL of ZNGs, filopodia appeared to be more numerous and arranged in an orderly manner along the cell membrane with respect to the control cells (Figure S1), in agreement with our previously report where zinc oxide-based nanomaterials induce the reorganization of actin cytoskeleton mainly in filopodia [38]. Filopodia are highly dynamic structures that the cells can use as sensors, to evaluate the surrounding environment stimulating a response through the activation of specific signaling pathways [72].

### 2.7. Intracellular ROS Detection in HaCaT Cells

**Figure 8:**
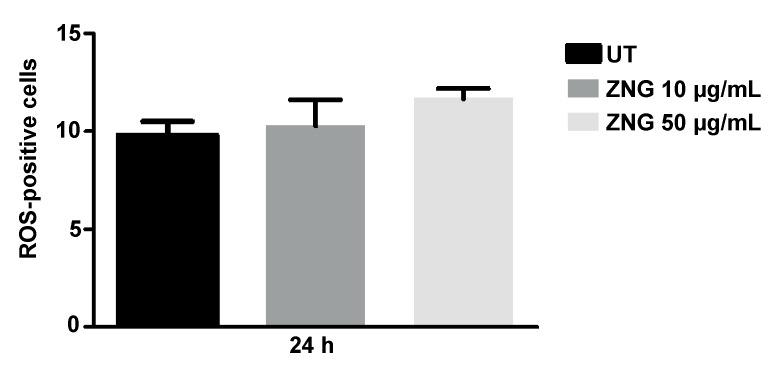
Intracellular ROS production of HaCaT cells after treatment with ZNGs or not (UT) for 24 h. Data are expressed as mean ± SD. Statistical analysis was performed by one-way analysis of variance (ANOVA) method coupled with the Bonferroni post-test. Values obtained are not significant.

ROS generation was next evaluated while using the dye H2DCFDA. HaCaT cells exposed or not to ZNGs at concentrations of 10 or 50 µg/mL for 24 h, showed a very slight non-significant increase in the number of ROS positive cells with respect to the control cells (Figure 8). This result, which indicates a very low toxicity of the ZNGs used in this in vitro system, is in agreement with the data reported for the ROS generation in the presence of ZnO-NR and ZnO-MR in HaCaT and MCF7 cells [38].

This finding could be explained by the thermal decomposition method used for the preparation of the ZnO utilized here that provides the release of very few zinc ions [73]. In fact, at high concentrations zinc ions are considered to be mainly responsible of the induction of oxidative stress, and, therefore, of the formation of ROS in the cells [74] whereas, the decrease of ZnO nanoparticles is responsible for a reduced toxicity, as demonstrated in rodent and zebrafish models [75].

### 2.8. MTT on ZNGs Coated Glass Slides

Many cytotoxic experiments have been carried out in vitro following the traditional approaches created for soluble nanoparticles [76]. In these conditions, nanomaterials tend to aggregate and sediment. To abolish or reduce these problems, many authors grow cells on nanomaterials-coated scaffolds, as recently reported by Lasocka et al. that demonstrated the biocompatibility of L929 cells that were grown on graphene-coated microscope glass slides [77].

**Figure 9:**
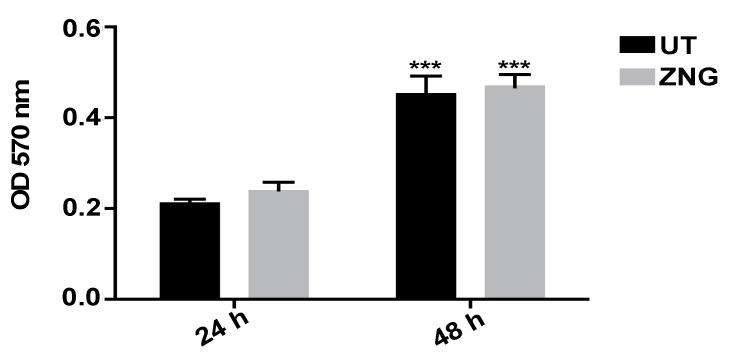
Analysis of cell viability, measured by the thiazolyl blue tetrazolium bromide (MTT) assay, of HaCaT cells grown on ZNGs coated glass for 24 and 48 h. Data are expressed as mean ± SD. Statistical analysis was performed by one-way analysis of variance (ANOVA) method coupled with the Bonferroni post-test (*** *p* < 0.001 compared to the untreated sample (UT)).

Based on this information, here, we have coated glass slides with ZNGs, and, on these, seeded the cells. To detect biocompatibility, we measured the optical density of living cells while using the MTT assay. After 24 h of growth on ZNGs coated glass slides, human keratinocytes showed a slight improvement in cell viability, also confirmed at 48 h in comparison with the cells seeded on uncoated glass (Figure 9). This result leads us to hypothesize that ZNGs are uniformly distributed and dispersed on the solid surface, indicating that ZNGs-coated substrates are a good scaffold for the cells, allowing for their use in several applications.

Our result is in agreement with that of Wang et al., which showed that ZnO/graphene oxide (GO) composites have a very low cytotoxicity on HeLa cells when compared to ZnO and ZnO nanoparticles (NPs) alone [78]. Moreover, nanohybrid materials composed by GO and chitosan showed a good biocompatibility regards murine fibroblast MC3T3 and human neuroblastoma SH-SY5Y cell lines [79] and L929 cells [80]. Thus, our findings demonstrate that the ZNG nanomaterials may be used as scaffold of different substrates for application in various fields, such as tissue recovery and engineering.

## 3. Materials and Methods

### 3.1. Materials

Glycerol anhydre, Trypan blue, anti-α-tubulin-FITC monoclonal antibody, Phalloidin-Tetramethylrhodamine B isothiocyanate (Phalloidin-TRITC), 4′,6-diamidine-2′-phenylindole dihydrochloride (DAPI), 2′,7′-Dichlorofluorescin diacetate (H_2_DCFDA), and thiazolyl blue tetrazolium bromide (MTT) were obtained from Sigma-Aldrich (Saint Louis, MO, USA).

### 3.2. Production and Morphology of Nanomaterials

Graphene nanoplatelets decorated with zinc oxide nanorods were produced and characterized as described in Chandraghari et al. [81]. ZNG is a hybrid material consisting of an array of vertically aligned ZnO-NR, directly grown on a graphene nano-platelet (GNP) flake. ZNGs were produced by a simple and cost-effective hydrothermal process, made up of two main steps, namely seed layer deposition and NRs growth. The first step involves the deposition of nucleation sites for the subsequent growth process. Briefly, the seed deposition consists in the preparation of an alcoholic solution of isopropanol and zinc acetate hexahydrates, in which a certain amount of graphene nanoplatelets (GNP) are suspended through magnetic agitation; the resulting suspension is then heated at 300 °C until thorough evaporation of the solvent. The second step consists in a hydrothermal process. This step is realized by immersing seed-coated GNPs in an equimolar aqueous solution of zinc nitrate dihydrate and hexamethylenetetramine for 4 h at 60 °C [81]. ZNGs morphology has been characterized through Field Emission-Scanning Electron Microscopy (FE-SEM) using a Zeiss Auriga (Carl Zeiss, Oberkochen, Germany) platform, operated at 5kV accelerating voltage (the complete set of micrographs is available at SNN-Lab, Sapienza University of Rome). Prior to imaging, no metallic coating was applied on the samples. FE-SEM micrographs reported in Figure S2A–C show ZNGs surface topography, while Figure S2D shows pristine GNPs, i.e., before the hydrothermal process. The average lateral dimensions of GNPs are in the 0.5 µm to 15 µm range, with thickness varying between 1 nm and 20 nm. ZnO-NRs have a diameter of 20–40 nm and a length of several hundred of nanometers (200–400 nm).

### 3.3. Fungal Strain and Culture Conditions

*Candida albicans* strain (ATTC 10231) was obtained from the American Type Culture Collection (ATCC) (Manassas, VA, USA). Fungal cells were routinely grown at 30 °C in YPD medium [1% yeast extract, 1% peptone, and 2% glucose-DIFCO (Difco, Becton Dickinson, Sparks, MD, USA)] under aerobic conditions. Strict anaerobic conditions were achieved while using OXOID Anaerobic Gas pack AN0010C (Oxoid Ltd., Basingstoke, Hants, UK), according to the manufacturer’s instructions.

To induce hyphal formation in vitro, *C. albicans* cells were grown at 37 °C in Spider medium [82].

To prepare a standard suspension for biofilm development, fungal cells were inoculated into YNB medium [(0.67% yeast nitrogen base w/o amino acids, 2% dextrose -DIFCO (Difco, Becton Dickinson, Sparks, MD, USA), and incubated for 18 h at 30 °C with agitation.

### 3.4. Cell Viability Assay of Yeast Cells

The survival of *C. albicans* following ZNGs treatment was evaluated by Colony Forming Units (CFU) assay. An aliquot of 5 × 10^7^ cells/mL from overnight culture was incubated at 30 °C for 24 h under gentle shaking with different concentrations of ZNGs suspended in H_2_O_dd_. The yeast cells were serially diluted and then spread on YPD agar plates. Yeast cultures grown under the same conditions without the addition of ZNGs served as controls. The survival rate was calculated according to the equation: [(CFU/mL) of exposed cells /(CFU/mL) of untreated sample] × 100.

### 3.5. Evaluation of Intracellular ROS Formation in Yeast Cells

The intracellular ROS production was detected while using the fluorescent probe H_2_DCFDA. *C. albicans* cells, grown overnight in YPD medium at 30 °C, were washed with phosphate buffered saline buffer (PBS), and incubated with different concentrations of ZNGs for 6 h and 12 h at 30 °C. After centrifugation, the cells were resuspended in a solution of 10 µM H_2_DCFDA for 30 min in the dark and washed twice with PBS. The fluorescent intensity was analysed with a Zeiss AxioVert 25 microscope (Carl Zeiss, Oberkochen, Germany). For each sample, the percentage of fluorescence was assessed in relation to the total cells per image.

### 3.6. Biofilm Assay

*C. albicans* biofilm formation was performed, as described by Thein et al. [83], with some modifications. Briefly, fungal cells were grown in 0.67% yeast nitrogen base w/o amino acids (YNB), supplemented with 100 mM glucose for 18 h at 30 °C. Afterwards, the cells were washed twice with PBS and resuspended to the concentration of 1 × 10^7^ cells/mL in PBS. 500 µL of the standard cell suspension (1 × 10^7^ cells/mL) were transferred into the wells of pre-sterilized, polystyrene, flat bottom 24-well plates (Falcon; Becton Dickinson, Lincoln Park, NJ, USA). The plates were incubated for 1.5 h at 37 °C in a static condition to promote the adhesion phase. After that, non-adherent cells were removed by washing the biofilms once with PBS and 1 mL of fresh medium (YNB and 100 mM Glucose) supplied with different concentrations of ZNGs (10, 50, 100, and 250 μg/mL) was added to selected wells. The plates were incubated for 24 h or 48 h at 37 °C in static and anaerobic condition.

### 3.7. SYTOX Green Staining

Vegetative cells of *C. albicans* was exposed for 6 h or 12 h to 10, 50, 100, and 250 µg/mL ZNGs, at 30 °C shaking gently. After that, the cells were washed twice with PBS and then stained with 1 µM SYTOX green (Thermo Fisher Scientific; Waltham, MA, USA) for 15 min at room temperature. Epifluorescence microscopy was carried out with a Zeiss AxioVert 25 microscope (Carl Zeiss, Oberkochen, Germany) fitted with a ×100 immersion objective and a standard filter set. Percentage of fluorescent cell was calculating relative to the total cells per image.

For biofilm staining, after the incubation for 24 h or 48 h, the wells were washed once with PBS and then stained with 1 µM SYTOX green for 15 min at room temperature. Finally, the plates were washed once with PBs and were observed to Zeiss AxioVert 25 microscope with the GFP filter (Carl Zeiss, Oberkochen, Germany).

### 3.8. Hyphal Growth on Solid Media

*C. albicans* cells from an overnight culture were diluted to OD_600_ 0.2 and spread on plates of solidified Spider medium supplemented with ZNGs. The plates were incubated for five days at 37 °C and the morphology of the fungal colony was photographed while using a digital camera.

### 3.9. Hyphal Growth in Liquid Media

Fungal cells grown in YPD medium for 18 h were diluted to obtain a cell concentration of 1 × 10^6^ cells/mL in Spider broth containing different concentrations of hybrid nanomaterials. Cells were incubated at 37 °C with agitation (250 rpm) for 2.5 h and then fungal cells were visualized by Zeiss AxioVert 25 microscope. The percentages of short and long hyphae were counted manually in relation to the total cells per image.

### 3.10. Cell Culture and Treatments

The human keratinocyte cell line HaCaT, spontaneously immortalized from a primary culture of keratinocytes [34], was cultured in Dulbecco’s Modified Eagle’s Medium (DMEM; Euroclone, Pero, MI, Italy), supplemented with 10% fetal bovine serum and antibiotics and was maintained in a humidified incubator with 5% CO_2_ at 37 °C.

The suspensions of ZNGs were freshly prepared in DMEM or DMEM with 4% glycerol followed by 30 min bath-sonication, and subsequently diluted.

### 3.11. Cell Viability and Proliferation of Human Keratinocytes

For the vitality assay, 2.5 × 10^4^ HaCaT cells were seeded on 24-well plates and cultured overnight. Subsequently, cultures were incubated or not with 10, 20, 40, and 50 µg/mL of ZNGs in DMEM or DMEM with 4% glycerol for 24 h (cell viability) or 10 and 50 µg/mL of ZNGs in DMEM with 4% glycerol for 24, 48, and 72 h (cell proliferation). Cells were detached by 0.05% trypsin-ethylenediaminetetraacetic acid (EDTA) solution and stained with Trypan blue for 2 min. The number of dead and viable cells was obtained by counting manually using a hemocytometer. Cell viability was expressed as total number of living cells. For cell proliferation, the number of cells was compared to that counted before treatment (t0). Values are representative of three independent experiments.

### 3.12. MTT Assay

HaCaT cells were seeded on glass slides coated with ZNGs and cultured for 24 or 48 h. Tetrazolium salts (MTT: 3-(4,5-dimethylthiazol-2-yl)-2,5-diphenyltetrazolium bromide, 5 mg/mL suspended in PBS), was added to each well and incubated for 4 h. The formazan crystals are extracted from the cells with a solubilizing solution (DMSO). An ELISA reader (Thermo Fisher, Phadia AB, Uppsala, Sweden). was used to measure the optical density at a wavelength of 570 nm and reference length 630 nm. The results were expressed as optical density values.

### 3.13. Intracellular ROS Detection in Human Keratinocytes 

HaCaT cells, which were grown on glass coverslips, were treated or not for 24 h with 10 or 50 µg/mL of ZNGs suspended in DMEM with 4% glycerol, and subsequently incubated with H_2_DCFDA, 10 μM, at 37 °C for 15 min. The detection of ROS was assessed at microscope by evaluating the number of positive cells for ROS production as compared to the total number of counted cells.

### 3.14. Immunofluorescence Microscopy

For immunofluorescence, 2.5 × 10^4^ HaCaT cells were seeded onto glass coverslips placed in 24-well plates and allowed to adhere. Subsequently, cells were treated for 24 h with 10 or 50 µg/mL of ZNGs in DMEM plus 4% glycerol. The cells were then fixed with 4% paraformaldehyde in PBS for 30 minutes, treated with 0.1 M glycine in PBS for 20 min, and with 0.1% Triton X-100 in PBS for additional 5 min to allow permeabilization. For the evaluation of the tubulin organization, the cells were incubated with the monoclonal antibody α-tubulin conjugated to fluorescein isothiocyanate (anti-α-tubulin-FITC) for 45 min. To analyze cytoskeletal actin reorganization, the cells were incubated with rhodamine-conjugated phalloidin (TRITC-phalloidin) for 45 min. The fluorescence signal was analyzed while using an Axio Observer inverted microscope, equipped with the ApoTome System (Carl Zeiss, Oberkochen, Germany).

### 3.15. Statistical Analysis

All data were expressed as mean values with the corresponding standard deviations (SD). To determine the significant differences, ANOVA analysis, followed by Bonferroni’s test, was conducted by using GraphPad Prism version 5.0 (San Diego, CA, USA).

## 4. Conclusions

The urgency of new compounds against *C. albicans* has been increased due to the failure of existing therapies and the development of drug resistance in clinical isolates. Germ tube and biofilm formation are important virulence factors that play an important role in the pathogenicity of this opportunistic fungus.

In this study, we investigated the anticandidal activity of ZNGs. We demonstrated that this nanomaterial not only affects the cell survival of *C. albicans*, but it also alters the pathogenicity of this fungus by shooting its virulence factor, like the elongation of germ tube and biofilm formation. In addition, our data indicate that the toxicity of ZNGs is partially associated to an increase of the ROS level in fungal cells. Furthermore, we tested the biocompatibility of this hybrid nanomaterial on HaCaT cells. We found that the exposure to ZNGs did not impair the proliferation and viability of keratinocyte cells, especially when ZNGs aggregation was reduced by adding glycerol to nanomaterial suspensions or by growing HaCat cells on glass slides that were coated with ZNGs.

In conclusion, our nanomaterial composed by ZnO nanorods-decorated graphene nanoplateles has proven itself a promising agent against *C. albicans* infections.

## Supplementary Materials

The following are available online at http://www.mdpi.com/2079-4991/8/10/752/s1, Figure S1: Immunofluorescence analysis of actin cytoskeleton of HaCaT cells by TRITC-phalloidin, after treatment with ZNGs for 24 h. White arrows indicate filopodia. Scale bar represents 20 µm; Figure S2: Field emission scanning electron microscopy (FE-SEM) images of (**a**), (**b**) and (**c**) ZnO-NRs-decorated GNPs (ZNGs) and (**d**) pristine GNPs.
