# Supplementary material for: Anti-Candidal Activity and In Vitro Cytotoxicity Assessment of Graphene Nanoplatelets Decorated with Zinc Oxide Nanorods
Source: Nanomaterials (Basel). 2018 Sep 21;8(10):752. doi: 10.3390/nano8100752 (PMC6215111; doi:10.3390/nano8100752)
Supplement: Supplementary file 1 [file nanomaterials-08-00752-s001.pdf]

Supplementary data

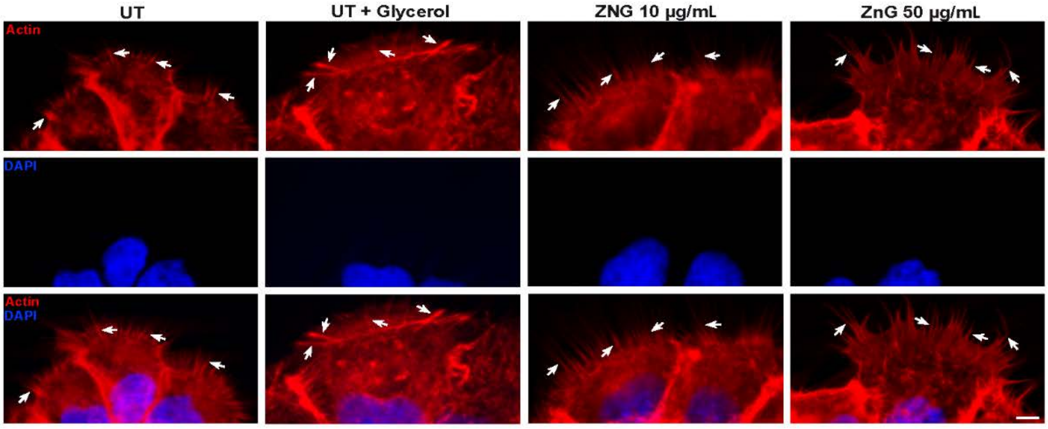

Supplementary Figure S1. Immunofluorescence analysis of actin cytoskeleton of HaCaT cells by TRITC-phalloidin, after treatment with ZNGs for 24 h. White arrows indicate filopodia. Scale bar represents 20 µm.

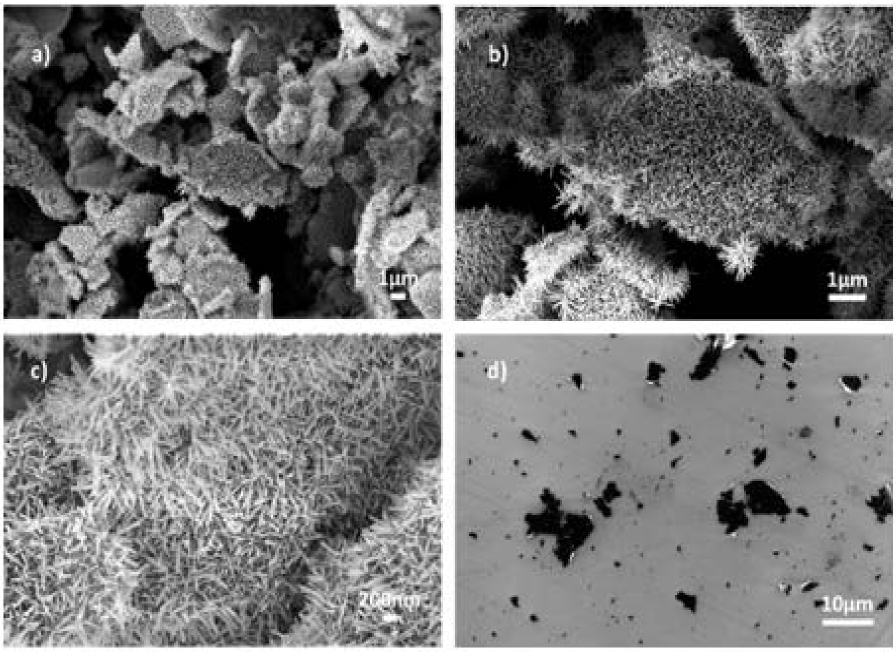

Supplementary Figure S2. Field emission scanning electron microscopy (FE-SEM) images of **a)**, **b)** and **c)** ZnO-NRs-decorated GNPs (ZNGs) and **d)** pristine GNPs.
